# Case Report: Aicardi-Goutières Syndrome Caused by Novel TREX1 Variants

**DOI:** 10.3389/fped.2021.634281

**Published:** 2021-04-28

**Authors:** De Wu, Liwei Fang, Ting Huang, Songcheng Ying

**Affiliations:** ^1^Department of Paediatrics, The First Affiliated Hospital of Anhui Medical University, Hefei, China; ^2^Department of Immunology, School of Basic Medical Sciences, Anhui Medical University, Hefei, China

**Keywords:** Aicardi-Goutières syndrome, Trex1, mutations, case report, type I interferons

## Abstract

TREX1 (three prime repair exonuclease 1) gene encodes DNA 3′ end repair exonuclease that plays an important role in DNA repair. Mutations in TREX1 gene have been identified as the cause of a rare autoimmune neurological disease, Aicardi-Goutières syndrome (AGS). Here, we report an AGS case of a 6-month-old Chinese girl with novel TREX1 variants. The patient had mild rashes on the face and legs, increased muscle tensions in the limbs, and positive cervical correction reflex. Cranial magnetic resonance imaging showed that there were patches of slightly longer T1 and T2 signals in the bilateral cerebral hemisphere and brainstem white matter, mainly in the frontotemporal lobe, together with decreased white matter volume, enlarged ventricles, and widened sulcus fissure. Total exon sequencing showed that the TREX1 gene of the child had mutations of c.137_138insC and c.292_293insA, which had not been reported before. In addition, elevated type I interferons were detected by using enzyme-linked immunosorbent assay in the patient's serum. Together, our study demonstrated that novel TREX1 variants (c.137_138insC and c.292_293insA) cause AGS for the first time.

## Introduction

Aicardi-Goutières syndrome (AGS) is a rare autoimmune neurological disease that is commonly observed in infants and young children (1). It is characterized by microcephaly, brain atrophy, intracranial calcification, lymphocytosis, and high levels of interferon α (IFN-α) in cerebrospinal fluid (CSF) (2). AGS is known to be a monogenic hereditary disease and can be divided into seven types according to the following seven pathogenic genes: TREX1 (AGS1), RNASEH2B(AGS2), RNASEH2C (AGS3), RNASEH2A (AGS4), SAMHD1 (AGS5), ADAR1(AGS6), and IFIH1 (AGS7) (2, 3). TREX1 gene encodes a potent DNA 3′ → 5′ exonuclease that clears cytosolic DNA to prevent aberrant inflammation and autoimmunity (4). Its mutations are associated with AGS, familial chilblain lupus, systemic lupus erythematosus, and retinal vasculopathy with cerebral leukodystrophy (5, 6). So far, the information about the diagnosis and therapy of the rare disease AGS caused by TREX1 is still less, although many pathogenic mutations of TREX1 have been identified (7). In this study, we report that novel TREX1 variants cause AGS.

## Case Report

### Routine Examinations

**Figure 1:**
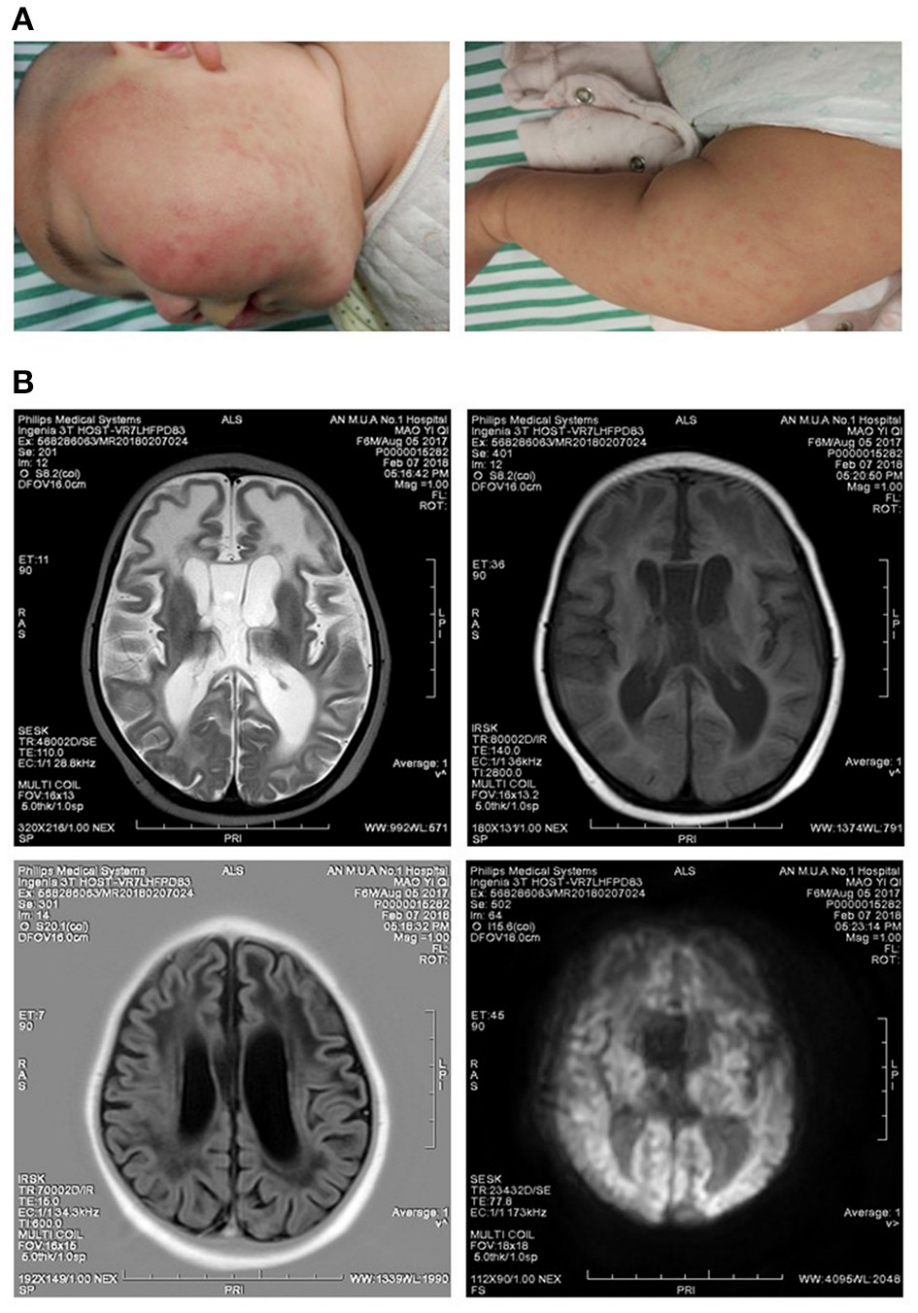
Clinical symptoms of the patient. **(A)** Mild rashes on the skins of the face and legs. **(B)** Slightly longer T1 and T2 signals were observed in the frontotemporal white matter, and the volume of white matter was decreased. Ventricles were enlarged, and sulci were widened.

The proband, female, 6 months old, was admitted to our hospital mainly because she could not laugh and displayed disabilities of eye tracking. The child was born at 39 weeks of gestation with Apgar score of 1–10 points, and her birth weight was 3,050 g. She had no history of asphyxia, hypoxia, rescue, and jaundice. Both parents are healthy and deny the family history of any genetic disorder. At the age of 3 months, the parents had found that the child could not laugh but paid little attention to it. At the age of 6 months, the child still could not laugh, and her eyes could not track the movements. Her admission physical examinations showed clear consciousness, normal spirit and nutrition, normal head circumference, normal complexion, mild rashes, and no head deformity (Figure 1A). There were no abnormalities in cardiopulmonary examination, no hepatosplenomegaly, and neck rigidity. Specialist examinations showed that the patient's ability of tracking sound was acceptable, but she did not pronounce babble sound. Her fists were clenched, and her thumbs adducted. She did not actively grasp and hold both hands. Her head can be raised in the prone position, but the erecting head was unstable when she was pulled up in the supine position. The muscle tension of her limbs was increased, and cervical correction reflex was positive (+). Slit lamp and fundus examinations yielded normal findings in both eyes. Development quotient of the girl was assessed by Gesell developmental schedules at the time of admission; all the function regions were lower than 2 standard deviations of normal children at the same age.

### Laboratory Tests

Glutamic oxaloacetic transaminase/aspartate aminotransferase 77 U/L, glutamic pyruvic transaminase/alanine aminotransferase 57 U/L, alkaline phosphatase 215 U/L, γ-glutamyl transferase 50 U/L, lactate dehydrogenase 427 U/L, and creatinine 22.9 mol/L, showing minor liver and kidney damage. Normal electrolyte, normal myocardial zymogram, immunoglobulin M 2.53 g/L, complement C4 0.41 g/L, and α-hydroxybutyrate dehydrogenase 314 U/L. Fecal routine plus occult blood test: negative. Five items of thyroid function test: negative. Reexamination of emergency blood routine test: lymphocyte percentage was 50.80% (range of normal value = 20–50%), lymphocyte absolute number was 4.53 × 10^9^/L (range of normal value = 1.1–3.2 × 10^9^). Torch series examination results showed that *Toxoplasma gondii*, herpes simplex virus 1 and 2, an rubella virus were negative, but human cytomegalovirus (HCMV) DNA was significantly higher than the normal range, and anticytomegalovirus lgG was positive. Ten immune series (hepatitis B surface antigen, anti–hepatitis B surface antibody, hepatitis B envelope antigen, anti–hepatitis envelope antibody, anti–hepatitis B core antibody, hepatitis B pre-S1 antigen, anti–hepatitis A antibody, anti–hepatitis C antibody, anti–human immunodeficiency virus antibody, and syphilis) were negative. Immunoglobulin and complement series were normal. Screening of blood and urine by tandem mass spectrometry showed no clinical significance.

### Auxiliary Examinations

**Figure 2:**
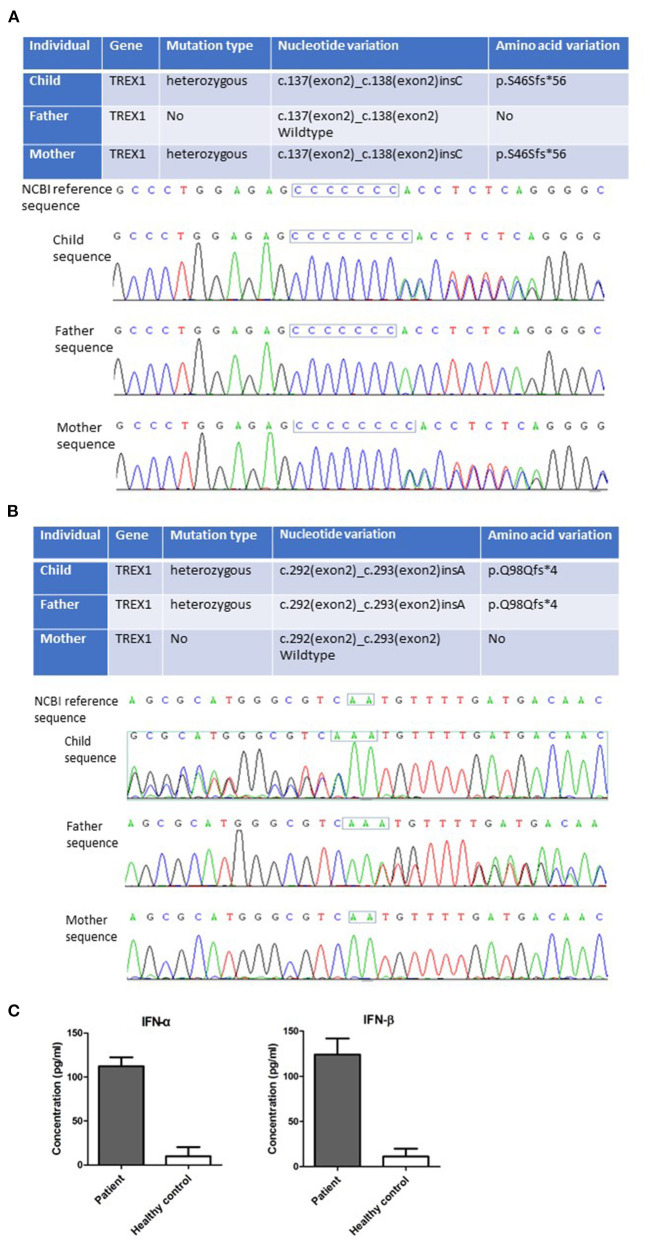
Novel TREX1 mutations and elevated type I IFN were found in the patient. New mutations of c.137 (exon2)_c.138 (exon2) insC **(A)** and c.292 (exon2)_c.293 (exon2) insA **(B)** in TREX1 gene of the patient were detected by Trio-based whole-exome sequencing. **(C)** Increased IFN-α and IFN-β levels in the sera of the patient were detected by ELISA. Statistically significant differences are expressed as ***P < 0.001.

An electroencephalogram was recorded during sleep, and no abnormalities were seen. Cranial magnetic resonance imaging (MRI) showed patches of slightly longer T1 and T2 signals in the bilateral cerebral hemisphere and brain stem white matter, mainly in the frontotemporal lobe; white matter volume was decreased, ventricles were enlarged, and sulci were widened. Median line was in the center, and there were no obvious abnormal flow vessels in the cranium (Figure 1B). These features indicated the patient had diffuse brain atrophy and delayed myelination, suggesting this disease was a type of toxic encephalopathy. With the consent of the parents, 2 mL venous blood of the child and that of their parents were collected, respectively, placed in the anticoagulant test tube, and sent to Beijing Quanpu Medical laboratory for Trio-based whole-exome sequencing. The results showed that the TREX1 gene of the child was mutated by c.137 (exon2)_c.138 (exon2) insC (Figure 2A) and c.292 (exon2)_c.293 (exon2) insA (Figure 2B). The mother TREX1 gene c.137 (exon2)_c.138 (exon2) insC was found (Figure 2A), but TREX1 gene c.292. (exon2)_C.293 (exon2) was normal (Figure 2B). The father TREX1 gene c.292 (exon2)_c.293 (exon2) insA mutation was observed (Figure 2B), whereas TREX1 gene c.137 (exon2)_c.138 (exon2) was normal (Figure 2A). According to the American College of Medical Genetics and Genomics rating guidelines, these variations were pathogenic. The parents of the proband were heterozygous carriers with normal phenotypes. However, the child was diagnosed as AGS1 by combining the clinical, imaging, and genetic findings. The enzyme-linked immunosorbent assay (ELISA) results showed that the serum concentrations of IFN-α and IFN-β were abnormally elevated in the patient (Figure 2C), consistent with the typical characteristics of AGS.

## Discussion

AGS was first discovered and described by Jean Aicardi and Francoise Goutières in 1984 (8). It was characterized by progressive encephalopathy, skin lesions, basal ganglia calcification, and raised IFN-α levels in the CSF (8). Later, this disease was known to be a single-gene hereditary disease. According to the pathogenic genes found, it was divided into seven subtypes: TREX1 (AGS1), RNASEH2B (AGS2), RNASEH2C (AGS3), RNASEH2A (AGS4), SAMHD1 (AGS5), ADAR1 (AGS6), and IFIH1 (AGS7) (2, 9). Loss-of-function mutants of AGS1–AGS6 genes were inherited in an autosomal recessive manner, and gain-of-function mutants of AGS7 gene were inherited in an autosomal dominant manner. In a few cases, specific pathogenic mutations in ADAR or TREX1 were inherited in the autosomal dominant manner (9). It was currently reported that TREX1 and RNASEH2B were most common among the seven pathogenic genes, accounting for 17 and 35%, respectively (10).

TREX1 gene was located at chromosome 3p21 and encoded DNA 3′ end repair exonuclease, which played an important role in DNA repair (5). TREX1 gene with loss-of-function mutations encoded the protein with no DNA exonuclease activity, resulting in the abnormal accumulation of nucleotides in the cytoplasm. The excessive accumulated nucleotides might be bound by the DNA sensor cGAS (the cyclic GMP-AMP synthase) and activated the IFN signal pathway through STING (stimulator of IFN gene), thus initiating the expression of type I IFN (IFN-I) and triggering the immune response (6). IFN-I played key roles in the antivirus and antitumor immune response, as well as immune regulation, but the excessive concentration of IFN-I could promote the antibody generation and lead to the development of autoimmune diseases (11). Furthermore, high levels of IFN-I might endanger the nervous system, thus producing AGS symptoms (12). Therefore, AGS was also known as an autoimmune disease with IFN-I abnormalities (2).

AGS had typical clinical manifestations (1, 2): (1) neurological symptoms: illness characterized by poor head control, dystonia, and pyramidal and extrapyramidal symptoms. Most patients had impairments of intelligence and movement abilities. A great number of patients lost language function, but a small portion still had good comprehension and expression ability. (2) Non-neurological symptoms: mainly chilblains. Approximately 40% of AGS patients suffered from chilblains, most of which were redness and swellings at the ends of hands and feet, ears, nose, and face, or even ulcers and necrosis, which were more common in winter. The child reported here had no obvious symptoms of chilblain at the time of treatment, but had erythema on the face and legs resembling rashes (Figure 1A).

AGS had characteristic imaging features (13): (1) Calcification. Patients showed cerebral calcification on CT scans and most MRI scans had hyperintense signals in T2-weighted images. As a diagnostic marker of AGS, calcification was mostly distributed in the patient's basal ganglia and white matter regions. (2) Abnormal white matter. The changes of MRI signal intensity mainly occurred in the white matter of the cerebral lobe, whereas the white matter around the ventricle, corpus callosum, and cystic and optic nerve was relatively less. (3) Brain atrophy and other changes such as corpus callosum dysplasia, putamen atrophy, striatal necrosis, etc. Brain MRI scan of the child reported in this article showed slightly longer T1 and T2 signals in white matter, mainly in the frontotemporal lobe, and volume of white matter decreased, indicating abnormalities in white matter. Consistent with symptoms reported by previous articles, the enlargement of ventricle and the widening of sulci in the child were discovered (Figure 1B), indicating the child had brain atrophy.

Human TREX1 protein contains an exonuclease domain on the N-terminus (242 amino acids) and a transmembrane helix on the C-terminus (72 amino acids), which is important for TREX1 localization to the endoplasmic reticulum or perinuclear space (14). AGS-related mutations of TREX1 are mostly located in the exonuclease domain (6). In this study, mutations of c.137_138insC and c.292_293insA of TREX1 gene in a 6-month-old child were identified by using total exon sequencing. These mutations resulted in the production of truncated TREX1 (p.S46Sfs^*^56 and p.Q98Qfs^*^4), which lacked the DNase function (Figures 2A,B). These truncated TREX1 mutations have not been reported before. In addition, high levels of IFN-α and IFN-β in the child's serum were detected by using ELISA (Figure 2C). Although the HCMV DNA of the patient on admission was positive, no viral DNA was detected from her umbilical cord blood at birth. The possibility of HCMV congenital infection leading to clinical symptoms could be ruled out. Importantly, the pathogenic TREX1 mutants were detected by Trio-based whole-exome sequencing. Together with the clinical phenotypes, this child was diagnosed as AGS caused by novel TREX1 variants.

At present, there is no specific and effective treatment for AGS. In theory, anti-IFN monoclonal antibody or inhibitors targeting the IFN signal pathway can be used for treating AGS (3, 15). It is gratifying that reverse transcriptase inhibitors and JAK inhibitor have been in clinic trails in treating AGS (information from www.clinicaltrials.gov). Currently, most strategies for treating AGS are aimed at patients with early onset. How to alleviate AGS symptoms and improve prognosis of patients who have suffered severe neurological damages need further researches.

## Data Availability Statement

The original contributions presented in the study are included in the article/Supplementary Material, further inquiries can be directed to the corresponding author.

## Ethics Statement

The studies involving human participants were reviewed and approved by the Ethic Committee of Anhui Medical University. Written informed consent to participate in this study was provided by the participants' legal guardian/next of kin. Written informed consent was obtained from the individual(s), and minor(s)' legal guardian/next of kin, for the publication of any potentially identifiable images or data included in this article.

## Author Contributions

DW, LF, and TH collected data, carried out the initial analyses, drafted the initial manuscript, reviewed, and revised the manuscript. SY conceptualized and designed the study, reviewed, and revised the manuscript. All authors approved the final manuscript as submitted and agree to be accountable for all aspects of the work.

## Conflict of Interest

The authors declare that the research was conducted in the absence of any commercial or financial relationships that could be construed as a potential conflict of interest.

## Abbreviations

TREX1: three prime repair exonuclease 1
AGS: Aicardi-Goutières syndrome
MRI: magnetic resonance imaging
IFN: interferon
CSF: cerebrospinal fluid
cGAS: cyclic GMP-AMP synthase
STING: stimulator of interferon gene.
